# Effects of 12 weeks of fascia knife release therapy in combination with exercise for treating neck and shoulder pain in adolescent table tennis players

**DOI:** 10.3389/fpubh.2025.1679219

**Published:** 2025-10-15

**Authors:** Li Yunqing, Zhang Shinian, Li Jie, Wu Xianfeng

**Affiliations:** ^1^Nanjing University of Chinese Medicine, Nanjing, China; ^2^Li Jie National Grassroots Famous Traditional Chinese Medicine Expert Inheritance Studio, Anqing, China; ^3^Anhui Institute of Sports Science and Technology, Hefei, China

**Keywords:** visual analog scale, cervical muscle endurance testing, neck disability index, fascia knife release therapy, functional exercise, neck and shoulder pain, adolescent table tennis player

## Abstract

**Background:**

We investigated whether fascia knife release therapy combined with exercise for treating neck and shoulder pain administered over a period of 12 weeks enhanced the visual analog scale (VAS), cervical muscle endurance test (CMET) score and neck disability index (NDI) score in adolescent table tennis players.

**Methods:**

This study adopted a prospective, exploratory design with a small sample size. The sample size was determined on the basis of the minimum requirements suggested by the relevant literature and feasibility considerations. Accounting for a potential 10% attrition rate, the study ultimately enrolled 40 adolescent table tennis players who were randomized to a control group or treatment group during the recovery period at the Anhui Sports Rehabilitation Clinic (Hefei, Anhui, China) in 2024. The subjects were divided into a medication group (*n* = 20, control group; CON) and a fascia knife release therapy combined with exercise group (*n* = 20, experimental group; EXP). A recovery exercise was applied to the control group for 12 weeks, whereas fascia knife release therapy combined with exercise was applied to the treatment group within the recovery empty period. The visual analog scale (VAS), cervical muscle endurance test (CMET) and neck disability index (NDI) were used to assess recovery before and after 12 weeks.

**Results:**

Comparisons of the visual analog scale (*p* = 0.854), cervical muscle endurance test (*p* = 0.393) and neck disability indices (*p* = 0.828) scores revealed no statistically significant differences between the CON group and the EXP group before the intervention. Compared with the control group, the EXP group presented significantly lower VAS (*p* = 0.001) and NDI (*p* = 0.001) scores following 12 weeks of fascia knife release therapy combined with exercise. There was a statistically significant increase in CMET in the treatment group (*p* = 0.001) compared with that in the CON group.

**Conclusion:**

Fascia knife release therapy combined with functional exercise enhances cervical muscle endurance (CMET) while significantly reducing pain (VAS) and functional disability (NDI) in adolescent table tennis players with neck and shoulder pain. Thus, this combined approach not only addresses immediate symptoms but also strengthens the muscles around the neck and shoulders, providing improved support and stability.

## Introduction

Musculoskeletal (MSK) pain is highly prevalent among children and adolescents, affecting up to 69% of those aged 8–16 years within a single school year (1). Approximately 25% of adolescents between the ages of 10 and 15 years experience MSK pain several days per week (2). Among those affected, six out of ten reported pain in the lower extremities, whereas three out of ten experienced spinal pain (3). MSK pain in young people is associated with diminished quality of life, school absenteeism, social withdrawal, anxiety, and sleep disturbances (4). Notably, neck and shoulder pain represents the most common type of musculoskeletal pain in adolescents. Neck pain, in particular, ranks as the eighth leading cause of disability globally among 15- to 19-year-olds (5). A questionnaire-based study involving 2,849 high school students in Shanghai, China, reported a neck and shoulder pain prevalence of 32.8% (6), which is often attributed to academic pressure among adolescents.

In recent years, with the pandemic of smartphones, the shift toward faster paced lifestyles and sedentary habits among adolescents has gradually led to an increase in neck and shoulder pain (7, 8). Neck and shoulder pain (NSP) is a common condition affecting adolescents (9). In Norwegian adolescents, frequent neck and shoulder pain has been reported in 20% of the population, indicating a notable prevalence within this age group (4). Pain in the neck, shoulder, and lower back is becoming more common in Finnish adolescents. Furthermore, this pain suggests a new disease burden of degenerative musculoskeletal disorders in future adults. Currently, reports of work- and sport-related neck and shoulder pain are increasing annually. Piano players have been found to have a higher neck disability index, indicating a greater risk of developing upper-body performance-related musculoskeletal disorders (10).

Similarly, sports that require repetitive neck and shoulder movements, such as table tennis, also contribute to this risk (11). Owing to the technical characteristics of table tennis, adolescent table tennis players are at greater risk due to their rapid growth and intense training regimens, which can lead to chronic pain, a decreased range of motion, and reduced athletic performance, thereby posing significant challenges to these young athletes. Thus, neck and shoulder pain in adolescent table tennis players is not only a common issue but also has serious implications for their health and athletic careers (12). Currently, research on the prevention of neck and shoulder pain in adolescents primarily focuses on pain management, education, physical exercise, ergonomic adjustments to daily furniture, physical therapy, medication (13), and, in severe cases, surgical intervention. However, these methods may not be ideal for young athletes because of potential side effects and prolonged recovery periods (14). Fascial scraping (also known as “fascial Gua Sha”) is an emerging alternative therapy for myofascial release. It involves the use of specialized tools to apply controlled pressure and friction to tense or restricted fascial tissues, thereby helping to reduce pain, improve mobility, and enhance tissue flexibility (15–17). This technique has gained increasing attention for its potential in preventing and treating fascial inflammation and related pain. However, there is limited research on treatment plans based on potential interventions for this specific population. Therefore, the purpose of this study was to evaluate the efficacy of fascia knife release therapy combined with functional exercise for treating neck and shoulder pain among adolescent table tennis players in the People’s Republic of China. By assessing the outcomes of a 12-week intervention, this research aims to provide practical strategies for the intervention and health management of neck and shoulder pain in adolescents. This approach will not only diversify treatment options but also help address existing research gaps in this field.

## Materials and methods

### Participants

The study was conducted in 2024 at the Anhui Sports Rehabilitation Clinic in Hefei, Anhui, China. This study adopted a prospective, exploratory design with a small sample size. The sample size was determined on the basis of the minimum requirements suggested by the relevant literature (18) and feasibility considerations. Accounting for a potential 10% attrition rate, the study ultimately enrolled 40 adolescent table tennis players. None of the participants were under medication, and all had at least 4 years of table tennis training along with a minimum of 3 years of competitive experience at the national level. The participants provided written informed consent after sufficient explanation of the experiment and an understanding of the possible adverse effects. They were assigned to a medication group (*n* = 20, control group; CON) or a fascia knife release therapy combined with function exercise group (*n* = 20, experimental group; EXP). There were no significant differences in physical characteristics among the groups before the intervention. This study was approved by the Ethics Committee of Anhui Sports Research Institute (AHTY-2024-01) in China. The participants in both groups were blinded to the specific treatment modalities and were informed that they would receive a 12-week course of therapy only prior to the experiment. To ensure the accuracy and validity of the tests, quality control measures were implemented for personnel involved in relevant processes such as testing, treatment, and data collection. The treatment for participants in both groups was primarily administered by the same team doctor, with another team doctor providing monitoring and assistance (Table 1).

**Table 1:** Participant demographic characteristics (*N* = 40).

Variables	CON	EXP
Number	20	20
Age (yr)	13.76 ± 1.60	12.66 ± 1.56
Height (cm)	172.92 ± 6.13	168.78 ± 10.29
Weight (kg)	59.23 ± 12.52	60.51 ± 7.18
Training time	4.19 ± 1.80	3.29 ± 1.35

CON, control group; EXP, experimental group.

### Study design

The study was conducted by the Sports Rehabilitation Therapy Team and designed as follows: 2 days of pretreatment assessment (including pain and range of motion measurements), 12 weeks of fascia knife release therapy combined with exercise intervention or medical treatment (or topical application of the NSAID Voltaren Emulgel), followed by 2 days of posttreatment assessment.

The participants in the control group received topical Voltaren Emulgel (diclofenac diethylamine emulsion, Novartis, Beijing, 20 g/tube) applied once per day for a total of 12 weeks. The dosage was adjusted according to the size of the painful area. Typically, 3–5 cm (or more) of the emulsion was applied per administration and gently rubbed into the skin to enhance absorption. All patients in the control group underwent therapy in the sport rehabilitation center and did not perform any exercise.

The participants in the experimental group underwent fascia knife release therapy combined with functional training. Fascia knife release therapy combined with functional training was administered once daily for a total of 12 weeks. (1) Fascia knife release therapy involved first identifying fascial adhesions and palpable nodules in painful areas via a C-shaped fascial scanning technique, followed by tool selection on the basis of the affected region’s size via an A-shaped shark-type fascial tool for smaller areas and a large M-shaped tool for broader regions. With patients in the prone position and target areas exposed, practitioners applied coupling gel before systematically performing myofascial release from the superior to the inferior direction on the sternocleidomastoid, platysma, surrounding soft tissues, scalene group, trapezius, and levator scapulae muscles while addressing the granular fascial points in the occipital musculature. During treatment, patients were instructed to perform alternating contraction–relaxation cycles of the affected muscles. Treatment duration continued until reduction or resolution of the granular fascial points, with the applied pressure maintained at patient-tolerant levels. Sessions were conducted once daily either from 9:00–10:00 a.m. or 2:00–3:00 p.m. in the rehabilitation training room under supervision. (2) Function exercises: The main content included the following: (I) Neck flexion and extension: Slowly lower your chin toward the chest (flexion), then gradually tilt the head backward to look at the ceiling (extension). (II) Lateral neck flexion: From an upright head position, the head should be tilted sideways toward one shoulder (attempting to approximate the ear to the shoulder), then it should return to neutral, then it should be repeated contralaterally. (III) While maintaining head alignment, the chin should be slowly rotated toward one shoulder until gentle stretching is felt, returning to the midline, and then it should be repeated on the opposite side. (IV) Shoulder Circling: Perform controlled circular motions with the shoulders initially completing forward rotations and then reversing to backward rotations. The participants participated in functional training (10:00–11:00 a.m. or 15:00–16:00 p.m.) during the recovery empty period for 10–15 min in the rehabilitation training room under supervision.

### Observation indicators

The research team assessed the efficacy of fascia knife release therapy in treating neck and shoulder pain in adolescent table tennis players, and several key outcome measures were utilized: the visual analog scale (VAS) for pain, cervical muscle endurance testing, and the neck disability index (NDI). These measures were taken at baseline, immediately after the completion of the treatment course, and at follow-up intervals to determine the immediate and long-term effects of the fascia knife release therapy on the neck and shoulder in adolescent table tennis players. Pain levels were assessed via the VAS, a widely recognized method for quantifying subjective pain intensity, and were measured according to the following procedure. The participants rated their pain on a scale from 0 (no pain) to 10 (worst possible pain) at baseline and at each follow-up visit. For the Cervical Muscle Endurance Test, Cervical muscle endurance was tested via a standardized protocol. The participants were asked to maintain a sustained cervical flexion or extension position for as long as possible without experiencing discomfort. The duration of the hold was recorded. The neck disability index (NDI) is a validated tool for assessing functional limitations associated with cervical spine disorders. It consists of a series of questions related to activities of daily living, work, and social activities. The participants completed the NDI questionnaire at baseline and at each follow-up assessment.

### Statistical analysis

All the statistical analyses were conducted via SPSS 26.0 (IBM Corp., Armonk, NY, United States). The data are presented as the means ± standard deviations. Differences between groups were compared via independent-sample *t* tests. The Bonferroni correction was applied to adjust for multiple comparisons, and a corrected *p* value < 0.05 was considered statistically significant. Effect sizes were interpreted via Cohen’s d, with values of 0.2 ≤ *d* < 0.5 indicating a small effect, 0.5 ≤ *d* < 0.8 indicating a medium effect, and *d* ≥ 0.8 indicating a large effect.

## Results

### Comparison of visual analog scale scores

**Figure 1:**
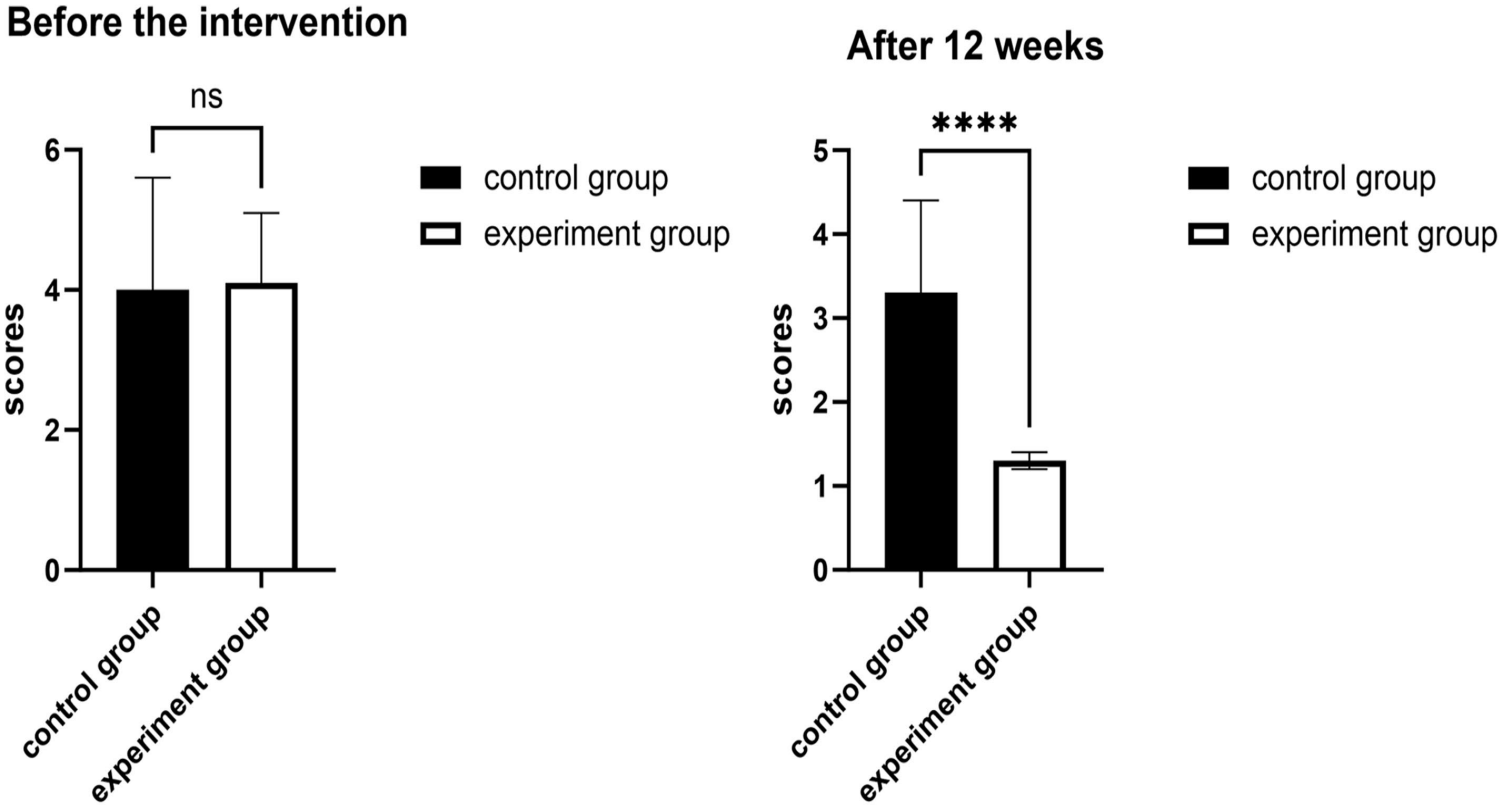
Effects of 12 weeks of fascia knife release therapy combined with exercise on VAS scores. The experimental group showed a significant difference compared to the control group after 12 weeks (ns: *p* > 0.05); The experimental group showed no significant difference compared to the control group after 12 weeks (****p < 0.01).

The comparison of the visual analog scale scores of the two groups revealed no statistically significant difference (*p* > 0.05, Cohen’s d = 0.07, 95% CI −0.95, 0.77) before the intervention (Figure 1). After the 12-week intervention, both groups’ VAS scores decreased, and the decrease in the treatment group was statistically significant (*p* < 0.05, Cohen’s d = 2.57, 95% CI 1.52, 2.53) (Table 2).

**Table 2:** Changes in visual analog scale scores in the two groups (*N* = 40).

Groups	Before the intervention	After 12 weeks
CON	4.0 ± 1.6	3.3 ± 1.1
EXP	4.1 ± 1.0	1.3 ± 0.1
*T* test	−0.186	8.502
*P* value	0.854	0.001

### Comparison of cervical muscle endurance test results

**Figure 2:**
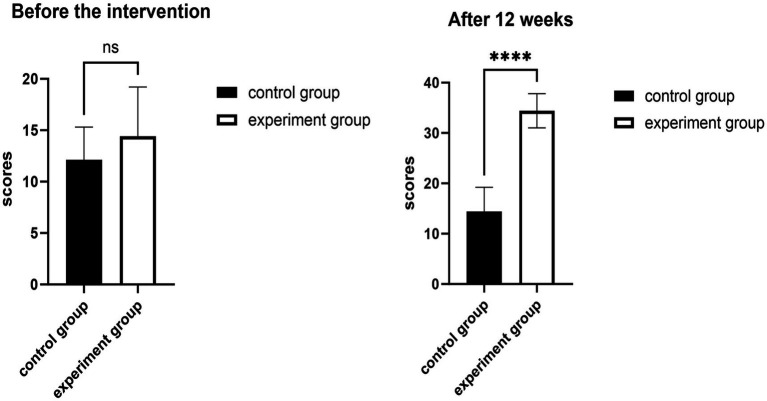
Effects of 12 weeks of fascia knife release therapy combined with exercise on CMET scores. The experimental group showed a significant difference compared to the control group after 12 weeks (ns: *p* > 0.05); The experimental group showed no significant difference compared to the control group after 12 weeks (****p < 0.01).

When the CMET scores of the two groups were compared, there was no statistically significant difference (*p* > 0.05, Cohen’s d = 0.23, 95% CI −2.95, 1.6) before the intervention (Figure 2). The CMET scores of both groups significantly increased after the 12-week intervention (*p* < 0.001, Cohen’s d = 4.81, 95% CI −22.84, 17.53), as shown in Table 3.

**Table 3:** Changes in cervical muscle endurance test results in the two groups (*N* = 40).

Groups	Before the intervention	After 12 weeks
CON	12.1 ± 3.2	14.4 ± 4.8
EXP	12.9 ± 3.7	34.4 ± 3.4
*T* test	−0.874	−22.229
*P* value	0.393	0.001

### Comparison of neck disability indices

**Figure 3:**
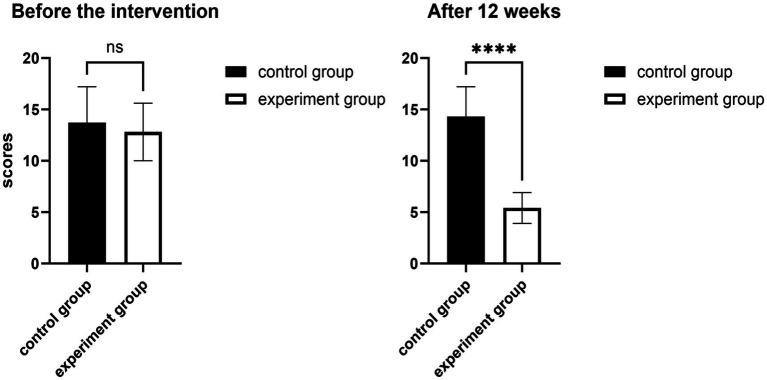
Effects of 12 weeks of fascia knife release therapy combined with exercise on NDI scores. The experimental group showed a significant difference compared to the control group after 12 weeks (ns: *p* > 0.05); The experimental group showed no significant difference compared to the control group after 12 weeks (****p < 0.01).

With respect to the comparison of the neck disability index before the intervention, there was no statistically significant difference (*p* > 0.05, Cohen’s d = 0.28, 95% CI −1.11, 2.9) between the CON and EXP groups before the intervention. After the 12-week intervention, the Neck Disability Index scores decreased significantly more in the EXP group than in the CON group (Figure 3). The difference was statistically significant (*p* < 0.05, Cohen’s d = 3.86, 95% CI 5.8, 10.41) (Table 4).

**Table 4:** Changes in neck disability indices in the two groups (*N* = 40).

Groups	Before the intervention	After 12 weeks
CON	13.7 ± 3.5	**14.3 ± 2.9**
EXP	12.8 ± 2.8	**5.4 ± 1.5**
*T* test	0.828	−11.078
*P* value	0.418	0.001

The bold values correspond to the Neck Disability Index (NDI) scores, which are presented in points. This index quantifies disability related to neck pain, with higher scores indicative of greater disability.

## Discussion

Neck and shoulder pain is a common condition affecting athletes, particularly those sports that involve repetitive neck and shoulder movements, such as table tennis (19, 20). In the context of adolescent table tennis players, shoulder pain is often due to poor sports technique and overuse. Intrinsic causes originate from the cervical spine, neck, and chest. Glenohumeral instability is a significant underlying mechanism for shoulder pain in table tennis players, whereas shoulder impingement syndrome is relatively uncommon (21). Moreover, myofascial trigger points are prevalent among university students and are associated with depression, anxiety, and stress. These findings indicate that researchers and clinicians should adopt a broader health perspective when addressing adolescents with neck and shoulder pain. It is important to consider the potential comorbidities and overall mental health of these young athletes (22). Therefore, clinicians and coaches should work together to ensure proper technique, monitor training loads, and provide psychological support to mitigate the risk of chronic pain and its associated comorbidities. Therefore, understanding these mechanisms is crucial for developing effective prevention and treatment strategies for neck and shoulder pain in table tennis athletes (23).

Currently, multimodal integrated strategies are widely recommended for the prevention and intervention of myofascial pain. These approaches typically include patient education, exercise therapy, behavioral correction, pharmacological treatment, and manual therapies, such as deep tissue massage and myofascial release (24, 25). Early intervention is often effective in alleviating symptoms; however, once Neck and shoulder pain progresses to a chronic stage, especially among adolescent athletes, treatment becomes considerably more challenging. In recent years, fascial release techniques have gained increasing attention as effective approaches for managing musculoskeletal pain. A study on fascial manipulation applied to patients with chronic shoulder pain demonstrated significant improvements in pain scores (*p* = 0.01) following treatment targeting specific fascial points in 18 participants (26). Another investigation reported that foam rolling, a form of self-myofascial release, significantly enhanced thoracolumbar fascia mobility by an average of 1.79 mm in healthy young adults (27). Furthermore, a randomized controlled trial evaluating the efficacy of myofascial release therapy combined with supervised exercise in individuals with subacromial pain syndrome revealed that after 4 weeks of combined treatment, patients experienced notable improvements in functional activities, a better balance between the upper trapezius and serratus anterior muscles, and enhanced dynamics of the periscapular muscles (28).

In addressing neck and shoulder pain among adolescent table tennis players, a multimodal approach combining targeted therapy and tailored exercise has shown considerable promise. Fascial knife therapy—a technique that utilizes specialized tools to apply controlled pressure and shear forces to the skin through scraping, pressing, and plucking maneuvers. This mechanical stimulation triggers biological responses such as fibroblast activation, proliferation, and differentiation, thereby facilitating soft tissue repair and regeneration. Therefore, this study was conducted over a 12-week period to investigate the effects of fascia knife release therapy combined with functional exercise on neck and shoulder pain in adolescent table tennis players. Before the experiment, the results revealed no significant differences between the control group and the treatment group in any of the cervical spine treatment efficacy evaluation indicators [VAS tests, *p* = 0.854 > 0.05, cervical muscle endurance test, *p* = 0.393 > 0.05, and neck disability index (NDI), *p* = 0.418 > 0.05]. These findings indicate that both groups started with similar levels of neck and shoulder pain, cervical muscle endurance, and functional disability. After the experiment, the results revealed a significant difference in all the cervical spine treatment efficacy indicators between the control group and the treatment group (VAS score, *p* = 0.00 < 0.05; Cervical Muscle Endurance Test score, *p* = 0.00 < 0.05; and NDI score, *p* = 0.00 < 0.05). Our study revealed that the frequent occurrence of neck and shoulder pain in table tennis players is closely related to cervical disc degeneration. This condition can lead to chronic pain, decreased range of motion, and reduced athletic performance, thereby posing significant challenges to these young athletes.

Therefore, it is hypothesized that the application of a fascial knife transmits pressure to the deep fascia and muscles. This pressure promotes the sliding of soft tissues such as the neck and shoulder muscles and fascia, enhances local peripheral circulation, and separates the adhered fascial layers. Together, these effects improve the tissue repair process, ultimately relieving muscle tension and reducing spasms. Moreover, a meta-analysis suggested that while specific exercises appear to be the optimal long-term strategy, a single session of nonspecific exercise seems more effective for immediate pain relief in patients with chronic neck or shoulder pain (29). Additionally, studies have shown that 4 weeks of supervised exercise combined with myofascial release can increase the range of motion of the shoulder, reduce pain, and improve the balance between the upper trapezius and serratus anterior muscles. This enhances periscapular muscle dynamics, thereby supporting functional activities in individuals with subacromial pain syndrome (30). Concurrently, supervised functional exercises targeting the neck and shoulders administered after fascial knife treatment can increase muscular endurance in these regions, further alleviating pain and enhancing overall athletic performance. Thus, this combined approach not only addresses immediate symptoms but also strengthens the muscles around the neck and shoulders, providing improved support and stability.

## Conclusion

On the basis of the experimental data analysis, this study revealed that fascial scraping combined with functional training improved neck and shoulder pain in young table tennis players. This method is simple and suitable for professional athletes as both a preventive and rehabilitative measure during routine training. These findings suggest that incorporating fascial release (scraping) into prevention and treatment plans for adolescent neck and shoulder pain is crucial.

Furthermore, this study has several limitations. The present study focuses primarily on short-term treatment outcomes, is constrained by sample size and study design, and features a limited follow-up period that lacks long-term tracking of patients’ rehabilitation progress. Although the combination of fascial dissection tools and functional rehabilitation exercises shows promising clinical benefits, its precise mechanisms of action remain incompletely understood—including how it inactivates myofascial trigger points in the neck and shoulder regions and facilitates the recovery of muscle strength and joint mobility. Additionally, this study used assessment tools such as the visual analog scale (VAS) for pain, the cervical range of motion, cervical muscle endurance testing, and the neck disability index (NDI), which rely heavily on patient self-reports and are susceptible to subjective bias.

To address these limitations, future studies should aim to expand sample sizes, extend follow-up durations, and include more diverse populations (e.g., swimmers and office workers). Moreover, we acknowledge that assessors were not totally blinded in this study due to practical constraints and randomized controlled trials (RCTs) should be conducted to evaluate long-term effects and incorporate biomechanical measures, along with other objective indicators. Such efforts will contribute stronger and more comprehensive evidence to support the prevention and management of neck and shoulder pain, particularly among adolescent populations.

## Data Availability

The datasets presented in this study can be found in online repositories. The names of the repository/repositories and accession number(s) can be found in the article/supplementary material.
